# Comparing Resident Procedures in Rural Versus Urban Emergency Departments

**DOI:** 10.7759/cureus.19989

**Published:** 2021-11-29

**Authors:** Nicholas I Carey, Joseph Hansroth, Hanna Davis, Brian Z Dilcher, Scott W Findley

**Affiliations:** 1 Emergency Medicine, West Virginia University School of Medicine, Morgantown, USA

**Keywords:** urban and rural communities, medical resident education, procedure training, rural hospital, emergency medicine resident

## Abstract

Background: Rural rotations can be a valuable experience for emergency medicine (EM) residents. To date, there has not been a retrospective cohort study comparing procedures performed at urban versus rural emergency departments (EDs).

Objectives: The purpose of this study was to compare procedures performed by EM residents in urban versus rural EDs, with the hypothesis that there will be no significant difference in the procedures performed.

Methods: A retrospective cohort study was conducted comparing procedures performed by second- and third-year EM residents based on medical chart review. The procedures were counted at three locations in West Virginia, including a small rural ED, a large rural ED, and a tertiary care ED. Procedure notes were collected from September 2018 to September 2019. The final analysis included nine months, as three months did not have residents at all locations. Eight procedures were standardized based on the number of procedures performed per 100 hours worked by residents. A comparison of total procedures and complex versus simple procedures was performed. A Kruskal-Wallis H test was performed to compare resident hours for procedures between each of the three locations. To compare each of the hospitals to one another separately, Mann-Whitney U tests were performed.

Results: The total resident hours worked included 1,800 at the small rural ED, 13,725.5 at the tertiary care ED, and 5,319 at the large rural ED. A p-value of 0.0311 for the Kruskal-Wallis H Test indicated a difference between at least two of the ED sites. A statistically significant difference exists (p-value = 0.0135) between the urban ED (95% CI: 0.15-0.62) and the large rural ED (95% CI: 0.54-1.53). There was no significant difference in complex versus simple procedures among the three locations (p-value = 0.4159).

Conclusions: When compared with the tertiary care ED, residents performed more total procedures at the large rural ED and similar total procedure numbers at the small rural ED when standardized for hours worked. There was no significant difference when comparing complex and simple procedures among the three locations.

## Introduction

There is a shortage of emergency medicine (EM) trained physicians in rural settings. In the last decade, the shortage has increased [1,2]. To add to the problem, rural physicians are closer to retirement age than those in an urban setting [1]. Exposure to rural experiences correlates with an increase in practicing in remote areas, and residents who participate in a rural emergency rotation are more likely to work in a rural area post-residency [3]. To prepare residents for the unique challenges faced in the rural emergency department (ED), resident training should incorporate rural experiences. The optimal rural rotation should expose residents to limited specialty consultation, limited on-site resources, and complex dispositions. This should not come at the expense of procedures performed, patient volume, and patient acuity.

In 2010, Wadman et al. compared the clinical experiences of five residents in rural versus urban emergency departments using self-reported resident procedures as one of the points of comparison [4]. Our study seeks to build off of this by comparing procedures performed by almost 30 residents, in three unique ED settings, retrospectively, using a shared electronic medical record (EMR) as data collection.

## Materials and methods

The primary means of data collection was based on the review of procedure notes in three unique environments within a connected academic hospital network: an academic tertiary care center, a large rural community hospital, and a small rural community hospital. All locations have a fast track staffed by advanced practice providers. The three hospitals have been on the same EMR since September 2018.

The academic tertiary referral center is a level 1 trauma center and a comprehensive stroke center with a 45-bed ED serving approximately 54,000 ED visits annually. The tertiary care center is considered urban based on the United States Department of Agriculture data, with a Rural-Urban Commuter Access (RUCA) code 1.0, and trains approximately 410 resident physicians and fellows through 50 programs. RUCA aims to standardize community resources and characteristics based on United States census data [5].

The large rural site is a level 4 trauma center with a 33-bed ED and an annual census of 58,000 ED visits. Every month, three to four EM resident physicians rotate through the department. The only other training program at the facility is a family medicine residency program. The hospital is located in an area with a RUCA code 4.0 and is considered micropolitan.

The small rural site is a federally designated critical access hospital with an eight-bed emergency department and an attached six-bed urgent care. The facility sees a combined 25,000 yearly visits with 12,500 of those presenting through the ED side. Three of the five regularly scheduled EM physicians at this facility are board-certified EM physicians, allowing residents to work at the site on a routine basis. There are no other training programs at this hospital. The community has a RUCA code 7.0 and is considered rural. Second- or third-year EM resident physicians can choose to participate in a rural emergency medicine rotation at this facility.

To collect our data, we performed an EMR screen and reviewed all second- and third-year emergency medicine residents’ notes at each facility between September 2018 and August 2019. First-year residents were not included, as they do not rotate at the small community site. The procedures were then standardized based on 100 hours worked by residents at each facility.

We performed a subgroup analysis to determine the frequency of simple versus complex procedures at each site (Table 1). For this analysis, we included all procedures occurring at greater than 0.5 occurrences per 100 resident hours at any location. The procedures were designated as simple based on the ability to be performed in the urgent care setting or complex if they would most likely require a staffed emergency department. A panel of practicing American Board of Emergency Medicine (ABEM) board-certified physicians then agreed that the procedures were grouped appropriately.

**Table 1:** Categorization of simple versus complex procedures and other procedures not included in the subgroup analysis. The other procedures included in total counts, but not occurring at greater than 0.5 occurrences per 100 resident hours, are as follows: foreign body removal (ocular, nasal, ear, and fish hook), chest tube, paracentesis, thoracentesis, arthrocentesis, and g-tube exchange.

Simple	Complex
Laceration Repair	Joint/Fracture Reduction
Splint Placement	Central Venous Access
Incision and Drainage	Lumbar Puncture
	Rapid Sequence Intubation
	Procedural Sedation

A Kruskal-Wallis H test was used to test for a statistically significant difference for both total procedures and complex versus simple procedures among the three locations. If a significant difference was found, Mann-Whitney U tests were performed to describe these differences. Statistical analysis was performed using the JMP® software (version 16.0.0, JMP Inc., Cary, NC, USA).

## Results

The total resident hours worked included 1,800 at the small rural ED, 13,725.5 at the tertiary care center, and 5,319 at the large rural ED. A p-value of 0.0311 for the Kruskal-Wallis H Test indicated a significant difference between at least two of the ED sites indicating the need for Mann-Whitney U tests (Table 2). A statistically significant difference exists (p-value = 0.0135) between the tertiary care center (95% CI: 0.15-0.62) and the large rural ED (95% CI: 0.54-1.53), favoring more resident procedures performed at the large rural ED (Table 3). There was no difference in total procedures between the tertiary care center and the small rural ED (p-value = 0.875) (Table 4). There was no significant difference in complex versus simple procedures among the three locations (p-value = 0.4159); therefore, Mann-Whitney U tests were not indicated (Table 5). The total number of procedures performed at the respective facilities are outlined in Table 6. 

**Table 2:** Comparison of total procedures per 100 resident hours worked among the three locations.

Tertiary Care Center ED	Large Rural ED	Small Rural ED	p-value
3.75	8.84	4.67	0.0311

**Table 3:** Comparison of total procedures per 100 resident hours worked (tertiary care center ED versus large rural ED).

Tertiary Care Center ED	Large Rural ED	p-value
3.75 (95% CI: 0.15–0.62)	8.84 (95% CI: 0.54–1.53)	0.0135

**Table 4:** Comparison of total procedures per 100 resident hours worked (tertiary care center ED versus small rural ED).

Tertiary Care Center ED	Small Rural ED	p-value
3.75 (95% CI: 0.15–0.62)	4.67 (95% CI: -0.058–-1.13)	0.875

**Table 5:** Simple versus complex procedures per 100 resident hours among the three locations. Kruskal–Wallis H Test: p-value = 0.4159

	Tertiary Care Center	Large Rural ED	Small Rural ED
Simple	1.25	3.85	3.06
Complex	2.50	4.98	1.61

**Table 6:** Total number of procedures at each emergency department prior to standardizing for hours worked. Procedures are separated by complexity. The total number of hours worked by residents is listed at the bottom.

	Tertiary Care Center	Large Rural	Small Rural
Complex Procedures			
Ocular Foreign Body	1	0	2
Joint/Fracture Reduction	18	50	4
Lumbar Puncture	31	29	10
Rapid Sequence Intubation/Intubation	80	37	4
Central Line	43	71	5
Electrical Cardioversion	3	3	0
Nerve Block	14	7	0
Chemical Cardioversion	4	0	0
Arthrocentesis	8	9	1
Procedural Sedation	82	26	1
G-Tube Exchange	6	4	0
Paracentesis	13	13	0
Transcutaneous Pacing	0	1	0
Pericardiocentesis	2	0	0
Arterial Line	23	3	0
Chest Tube	9	9	0
Ear Foreign Body	1	0	2
Lateral Canthotomy	0	1	0
Dialysis Catheter	1	0	0
Pigtail Insertion	3	2	0
Thoracentesis	1	0	0
Simple Procedures			
Nasal Foreign body	2	1	0
Laceration Repair	123	105	40
Splint Placement	22	60	2
Incision and Drainage	22	19	11
Nail Removal	0	1	1
US IV placement	1	19	0
Fish Hook Removal	0	0	1
Total Hours	13725.5	5319	1800

## Discussion

A recent national workforce study showed that 92% of emergency physicians practice in urban areas despite 35% of the country’s 5,198 hospitals being registered as rural [1,6]. Casaletto et al. outlined a guide for program directors to implement rural ED resident rotations [7], and Talley et al. showed that residents with a rural rotation are more likely to practice in this environment [3]. With an aging rural EM workforce, increasing recruitment and retention of young physicians in rural areas will be necessary to continue providing high-level care in the future to meet workforce needs [1]. Residents rotating in rural locations learn valuable skills such as allocating resources, working with limited specialty backup, and identifying appropriate patient transfers. Although valuable, this skill acquisition should not come at the cost of compromising patient volume, acuity, and procedures that may be encountered in more urban settings.

In 2010, Wadman et al. compared rural versus urban ED rotations and found no significant difference in patients seen per hour but showed mixed results on procedure frequencies [4]. One limitation of this study was the small sample size and the self-reporting of procedures performed. To expand on prior research, we compared procedures across multiple sites in West Virginia using a larger pool of resident doctors and recording procedures based on EMR documentation. The primary outcome was to evaluate procedure rates in multiple practice environments (tertiary care, large rural, and small rural).

The primary outcome of this study shows that procedures, when standardized based on hours worked, are found to be highest at the large rural site but are consistent regardless of the practice environment. One reason for this may be the unopposed nature of small hospitals; in large hospitals with other procedure-based training programs, procedures get divided between emergency medicine residents and consulting service residents [8]. However, in hospitals with limited consultants, EM providers must be relied upon to perform these procedures or else must transfer the patient to a referral center. The procedures measured in this study are core to emergency medicine, and the majority are required for graduation from an EM residency program [9]. It has been shown that when annual patient volumes are greater than 15,000, there is no difference between rural and urban patients seen per physician hour [10]. This data argues that procedures performed in rural areas occur at a similar rate to urban areas, challenging the notion that sick patients are not seen in small hospitals and skill decay will occur in these practice environments.

The rural hospitals studied were of larger volumes than many rural hospitals across the country. The two rural hospitals included in this study both still have challenges of being geographically distant from a higher level of care with weather and terrain that can make air or land transport difficult. In-house resources and specialty coverage are also rarely rapidly available and may have unpredictable schedules, especially on nights, weekends, and holidays.

We do believe that there is a volume threshold where skill decay will occur, but it is likely at a lower threshold than commonly thought, especially in the context of the COVID-19 pandemic, based on emerging surgical literature [11,12]. The exact census for this skill decay threshold is not known but could be a topic for future research. An internal review of all our single-coverage critical access hospitals in our network indicates roughly 10% of daily volumes present between midnight and 7 am. Residents included in this analysis were not restricted from working night shifts at our small rural sites. After reviewing this daily trend, residents are no longer scheduled to work during hours when average volumes are less than 1.0 patients per hour. If anything, this will lead to increased procedure numbers in the small rural facility.

There has been a discussion of emergency medicine and primary care partnership to meet the shortage of rural EM physicians [13,14]. As the national emergency physician workforce study showed, only 52% of physicians in large rural and 37% in small rural are emergency medicine trained. Many of the remaining providers are family medicine trained [1]. While some of the procedures performed in the ED are required during family medicine residency, their graduation requirements focus on other aspects of training, and the specific number of measured procedures is less than that of EM [15]. In addition, many of the complex procedures studied here are beyond the scope of advanced practice providers working independently. This study demonstrates that there is no significant difference between procedures performed in rural versus urban departments, and therefore, these appear to be viable rotation options for EM resident training.

This study can be used as a model for data collection. The department of Emergency Medicine at West Virginia University has continually been increasing the number of rural locations through which residents can rotate. These sites all use the same EMR, and as the number of resident hours worked accumulates, there will be more power to future studies. Other locations can also provide information on rural facilities with varying degrees of annual volumes. Collaborative efforts among residency programs in other geographic areas that offer rural rotations would also be of value to compare rural versus urban experiences. The data could be used to identify the strengths and weaknesses of rural rotations and improve the clinical experience in the future.

## Conclusions

This study shows that there is no significant difference in procedures performed by residents at both small rural and large rural emergency departments each compared with urban ED. A clinical rotation in the rural setting can give residents exposure to rural emergency medicine without compromising procedural experiences they would otherwise have in an urban ED.
